# Salivary pH, uric acid and C - reactive protein in conventional versus chitosan-nanoparticle denture wearers

**DOI:** 10.6026/973206300214111

**Published:** 2025-11-15

**Authors:** Chanchal Anand, Nivedita Dixit, Monika M, Srashti Singh, Manisha Gaurav, Shyamli

**Affiliations:** 1 Department of Prosthodontics and Crown & Bridge, Institute of Dental Sciences, Bareilly, Uttar Pradesh, India

**Keywords:** chitosan nanoparticles, complete denture, saliva, pH, uric acid, C-reactive protein, biomarkers, inflammation.

## Abstract

Edentulous patients wearing complete dentures often face poor denture hygiene and mucosal inflammation. Incorporating antimicrobial agents such as chitosan nanoparticles (CNPs) into dentures may improve the oral environment. In this study, salivary pH, uric acid and C-reactive protein (CRP) were compared between conventional dentures and CNP-incorporated dentures in edentulous patients. After one month of use, CNP\ dentures significantly increased salivary pH and uric acid while showing a non-significant decrease in CRP. Thus, we show that CNP incorporation enhances oral health by improving salivary parameters and reducing inflammatory responses.

## Background:

The oral cavity is a highly dynamic ecosystem where saliva serves as a central component in maintaining tissue integrity, regulating microbial balance and ensuring prosthesis comfort [1]. In completely edentulous individuals, the insertion of complete dentures restores essential functions such as mastication, speech and aesthetics; however, these prostheses also significantly influence the salivary environment [2]. Conventional acrylic dentures, though widely used, often act as reservoirs for microbial colonisation, particularly *Candida albicans* and *Streptococcus mutans*, thereby predisposing patients to mucosal inflammation and denture stomatitis [3, 4]. Previous studies have reported changes in salivary flow rate, pH and microbial composition in denture wearers, underscoring the need to address prosthesis-related alterations in the oral milieu [5, 6-7]. Recent advancements in biomaterials have focused on the modification of denture base resins to counteract microbial adherence and improve oral tissue health. Chitosan, a naturally derived polysaccharide, has attracted attention due to its excellent biocompatibility, biodegradability and antimicrobial properties [8, 9]. When processed into nanoparticles, chitosan demonstrates enhanced biological activity as its nanoscale dimensions and positively charged amine groups enable stronger surface interactions with negatively charged microbial cell membranes. This interaction disrupts microbial cell walls, inhibits biofilm formation and reduces colonisation of pathogenic species [10, 11]. Furthermore, chitosan nanoparticles (CNPs) possess intrinsic antioxidant potential, which can contribute to the reduction of oxidative stress in the oral environment [12]. The incorporation of CNPs into denture base materials therefore represents a novel approach to improving prosthesis-related oral health outcomes. Saliva is widely regarded as a valuable diagnostic fluid due to its non-invasive accessibility and its ability to reflect both local and systemic health conditions [13]. Among its measurable biomarkers, salivary pH is a critical determinant of microbial balance and tissue health, while uric acid represents the predominant antioxidant that neutralises reactive oxygen species [14]. Conversely, elevated salivary levels of C-reactive protein (CRP), an acute-phase inflammatory marker, are indicative of mucosal inflammation and systemic immune responses. Alterations in these biomarkers provide insight into the oral environment of denture wearers, reflecting microbial shifts, oxidative stress and inflammatory status. Considering these perspectives, the present study was designed to evaluate and compare salivary pH, uric acid and CRP levels in completely edentulous patients rehabilitated with either conventional acrylic dentures or chitosan nanoparticle-incorporated dentures. Therefore, it is of interest to assess whether the integration of CNPs into denture base resin can positively modulate the salivary environment and contribute to improved mucosal health.

## Materials and Methods:

## Study design and participants:

This *in vivo* experimental study included thirty complete denture-wearing subjects who visited the Out-Patient Department of the Department of Prosthodontics and Crown & Bridge of Institute of Dental Sciences, Bareilly, Uttar Pradesh, for refabrication of their complete dentures. Patients with systemic disease, those taking medications that affect salivation, or those with poor compliance were excluded. Patients were well-informed before any involvement in the study. Proper consent forms were taken from patients, duly signed by them, informing them about the procedure carried out during the study. All subjects underwent a comprehensive medical history taking and oral lesion examinations were carried out.

## Study protocol:

Saliva samples were collected from the subjects in the following phases:

[1] 1st sample collection: The first sample was collected when a patient visited the Out-Patient Department of the Department of Prosthodontics and Crown & Bridge of Institute of Dental Sciences, Bareilly, with a complaint of an unsatisfactory previous denture and wanted the replacement of the same.

[2] 2nd sample collection: The second salivary sample was collected from the patient after a month of wearing a chitosan nanoparticle-incorporated complete denture.

## Methodology of collection of salivary samples:

After rinsing the mouth 3-4 times with water, unstimulated saliva was then allowed to pool on the floor of the mouth for about two minutes. Using the passive drooling method, 2 mL of whole saliva was then collected into ice-chilled polypropylene vials. Samples contaminated with blood were discarded. The date and time of collection were recorded and all the samples were immediately stored at -20°C to prevent bacterial growth until further biochemical analysis.

## Clinical steps:

[1] The first salivary sample was collected from the patient on the first day of visit.

[2] Denture Fabrication and Chitosan Incorporation.

Complete dentures were fabricated using conventional heat-cured polymethyl methacrylate (PMMA) following standard clinical and laboratory protocols. Chitosan nanoparticles were incorporated into the PMMA resin at a concentration of 5% by weight. The nanoparticles were accurately weighed using a digital analytical balance (Figure 1 - see PDF) and thoroughly blended with the PMMA polymer powder to ensure uniform dispersion (Figure 2 - see PDF).

This modified polymer was then mixed with the monomer (methyl methacrylate) in the recommended powder-to-liquid ratio and allowed to reach the dough stage-a non-sticky, workable consistency suitable for packing. The resulting dough was carefully packed into the prepared denture molds. The flasks were closed, clamped and subjected to the standard long curing cycle (at 75°C for 6 hours, then 100°C for 1 hour) in a thermostatically controlled water bath. After polymerization, the dentures were deflasked, trimmed, finished and polished. Final prostheses were checked intraorally for retention, stability, occlusion and esthetics and delivered to the patient. The second salivary sample was collected from the patient after a month of wearing chitosan nanoparticle-incorporated complete denture.

## Analysis of salivary samples:

[1] pH was then measured using a digital pH meter.

[2] Uric acid was assessed via an enzymatic colorimetric assay.

[3] CRP was estimated using high-sensitivity ELISA kits.

## Statistical analysis:

Data were entered on a Microsoft Excel spreadsheet and imported into Statistical Package for Social Sciences (SPSS) version 23 for statistical analysis. The data was presented in mean and standard deviation. A one-way ANOVA test was performed to find significant differences in different variables between the groups. Statistical test: A paired t-test was employed to find significance. A p-value less than 0.05 were considered statistically significant and a p-value less than 0.001 were considered statistically highly significant.

## Results:

The analysis of salivary biomarkers before and after the incorporation of CNP dentures revealed notable changes. Table 1 (see PDF) displays the descriptive statistics for the biomarkers pH, uric acid, and CRP. Pre-incorporation, the mean pH was 6.34 (SD = 0.77), uric acid was 4.08 (SD = 1.38), and CRP was 3.47 (SD = 4.59). Post-incorporation, pH increased significantly to 6.79 (SD = 0.72), uric acid rose to 4.5 (SD = 1.53), while CRP decreased slightly to 3.36 (SD = 4.25). Figures 3 (see PDF) and 4 (see PDF) illustrate the changes in salivary biomarker levels before and after incorporating the CNP dentures, respectively. Pre-incorporation levels show a baseline for comparison, while post-incorporation measurements indicate a slight improvement in pH and uric acid levels, with a minimal reduction in CRP. The observed increase in pH and uric acid post-incorporation of CNP dentures is statistically significant, suggesting a potential benefit of these dentures in influencing salivary composition, though the change in CRP was not statistically significant.

## Discussion:

The findings of this study demonstrate that incorporation of chitosan nanoparticles (CNPs) into complete dentures can beneficially modulate salivary biomarkers in edentulous patients. A significant rise in salivary pH was observed after the use of CNP-modified dentures, indicating enhanced buffering capacity and suppression of acidogenic microbial activity. This change is clinically relevant, as acidic environments in denture wearers are typically associated with increased colonisation of *Candida albicans* and other acid-producing bacteria, leading to mucosal inflammation and denture stomatitis [3, 4-5]. By maintaining a more alkaline oral environment, CNPs appear to reduce the risk of microbial overgrowth and associated complications [6, 7]. Salivary uric acid, the principal antioxidant in saliva, also showed a significant increase following CNP denture insertion. This elevation suggests reinforcement of antioxidant defence mechanisms, which may help counteract oxidative stress in the oral cavity. Oxidative imbalance has been implicated in mucosal irritation and prosthesis intolerance [8, 9]. The antioxidant potential of CNPs, owing to their free radical scavenging and metal-chelating properties, may therefore contribute to better mucosal resilience and comfort in denture wearers [10, 11-12]. CRP levels, although not significantly reduced, showed a mild downward trend after one month of CNP denture use. This suggests a possible anti-inflammatory effect, likely mediated by decreased microbial load and reduced oxidative burden. The observed negative correlation between salivary pH and CRP further highlights the role of a more alkaline oral environment in lowering inflammatory activity [13, 14-15]. Even though the reduction in CRP was not statistically significant, the trend indicates potential for long-term benefits with extended use of CNP dentures [16]. Overall, the results suggest that CNP incorporation into denture bases contributes to a more stable and healthier oral biochemical environment. The combination of increased pH, enhanced antioxidant activity and reduced inflammatory tendency presents a promising strategy for preventing denture-related mucosal problems. However, the study's limitations-such as small sample size, short follow-up, absence of microbial quantification and single-centre data-must be acknowledged. Long-term, multicentric clinical trials with larger populations, integrated microbial analysis and comparisons with other bioactive denture modifications are warranted to confirm and expand these findings.

## Conclusion:

Incorporation of chitosan nanoparticles into complete dentures significantly improved salivary pH and uric acid levels and showed a trend toward reduced CRP levels. Thus, we show the potential of CNPs to improve the oral environment, offering antimicrobial and anti-inflammatory benefits. However, further studies with larger samples and longer follow-up are warranted.
